# Beijing cough: a case report

**DOI:** 10.1186/1757-1626-1-3

**Published:** 2008-05-12

**Authors:** Richard Smith

**Affiliations:** 1 Editor-in-Chief, Cases Journal, BioMed Central, Middlesex House, 34-42 Cleveland Street, London W1T 4LB, UK

## Abstract

**Case Presentation:**

Upon arrival in Beijing, China from London, England in 2008 a previously healthy 56-year old man developed a regular spasmodic cough for the duration of his stay in the city.

## Case presentation

I'm a 56-year-old male who doesn't smoke but drinks about 30 units of alcohol a week mostly in the form of red wine. My current job is best described as a health manager, and I'm very rarely ill. In 25 years working for the BMJ Publishing Group I missed no more than a handful of days. Similarly in six years at university I don't remember missing a day, and in seven years at school I missed one day. I haven't seen my GP for at least three years, although I can't help thinking I should do.

I've had no serious illnesses, although I did have moderately severe hay fever between the ages of 15 and about 40. It then disappeared. Sometimes after running I have developed a wheeze, which has never distressed me and usually disappeared within 20 minutes. Between being 20 and 30 I smoked the occasional cigarette and a fair few "joints" – tobacco and marijuana.

I flew to Beijing from London on British Airways on New Year's Day 2008, arriving at 7 am Beijing time on 2 January. The night before I had drunk very little because I was driving. I wasn't in a smoky atmosphere, and I went to bed in London around 2 am. I flew economy class and it was a very uneventful flight. I was struck by the emptiness and cleanliness of Beijing airport.

I was through the terminal very quickly and was driven into the city. As I was being driven I noticed the "fogginess" of the atmosphere. I didn't know how much was atmospheric and how much pollution. At some point early on I noticed that I could smell and almost "taste" the air – even in my hotel room. The smell was of coal. Arriving at my hotel I had breakfast – mostly Chinese rather than Western food – and then went to bed for a couple of hours. (Later I read that Beijing had some of its highest levels of pollution ever at the end of December and beginning of January. It was reported as "level five," meaning that old people and young children should not go out.)

In the afternoon I took a walk of perhaps two miles, again noticing the fogginess. It was cold, barely above freezing. I then worked in my hotel room for several hours before going round the corner for a meal and the going to bed around 10. When I woke in the night I could "taste" the air – even in the hotel, which was air conditioned.

The next day – 3 January – I worked in the hotel for much of the day but also went out for a walk. On the day after that I went with friends to the Forbidden City, where the fogginess was very obvious. We were there for several hours, and afterwards went to a Peking Duck restaurant and to a show.

It was on this day that I began to cough. I didn't feel at all ill, and I coughed nothing up. The next day we met with Chinese colleagues, had a series of meetings with them, and then flew to Shanghai with them. Shanghai seemed less polluted – both visually and by smell and taste – than Beijing, and it was warmer, although still only a few degrees above freezing at night.

My cough became stronger. It would come in spasms. During our many meetings I would suddenly dissolve into a spasm of coughing. A spasm might last 90 seconds. I would drink water – and that would help. A spasm might come every 10 minutes. I didn't feel ill, and I didn't cough up anything. My throat did feel irritated, but I didn't have what I would call a sore throat.

I didn't cough every 10 minutes all day long, but I would be coughing very regularly – perhaps 80 times a day. At night I would cough while falling asleep (which rarely takes me than two minutes), but I wasn't as far as I know awoken by my coughing. When I did wake, however, I might have four or five spasms of coughing. I'd cough heavily when I woke in the morning – at perhaps two-minute intervals for 20 minutes.

On Monday I met an Australian who visits Beijing regularly, and he said to me: "You've got the Beijing cough. I get it regularly, but it usually disappears as you fly home."

That's not what happened to me. My cough continued until I flew home to London eight days after arriving in Beijing, and it was still with me as three days after returning I flew to Boston and on to San Diego. In the next 10 days I visited Tijuana, El Paso, Juarez, Guatamala City, and Minneapolis – coughing all the way. But my cough was fading – and it was finally gone about two weeks after I left Beijing.

I didn't consult a doctor, but friends offered me antihistamines, antibiotics, cough mixtures, and even steroids. I didn't take anything.

Could this have been simply an upper respiratory tract infection? I don't think so. I never had a sore throat, never felt ill, and never coughed up anything.

I'm probably returning to Beijing next week. I'll feedback on whether I get a cough again. It would be interested to hear of the experiences of others who have visited Beijing.

## Consent

Written informed consent was not sought for publication of this case report since the author is the subject.

## Competing interests

I am the Editor-in-Chief of *Cases Journal*. I declare no other competing interests.

